# Autophagy Modulates Immunogenic Cell Death in Cancer

**DOI:** 10.3390/cancers18020205

**Published:** 2026-01-08

**Authors:** Maiko Matsushita, Miyu Moriwaki

**Affiliations:** Division of Clinical Physiology and Therapeutics, Faculty of Pharmacy, Keio University, Tokyo 105-8512, Japan

**Keywords:** autophagy, immunogenic cell death, damage-associated molecular patterns, tumor microenvironment, multiple myeloma

## Abstract

Immunogenic cell death (ICD) is a subtype of regulated cell death characterized by the spatiotemporally coordinated emission of damage-associated molecular patterns (DAMPs), such as calreticulin (CALR), ATP, and high-mobility group box-1 (HMGB1), which collectively prime tumor-specific T-cell responses. Autophagy, a lysosome-dependent catabolic process, is increasingly recognized as a key modifier of antitumor immunity and the tumor microenvironment (TME). In preclinical models, autophagy can not only promote ICD by sustaining endoplasmic reticulum (ER) stress, eukaryotic translation initiation factor-2α (eIF2α) phosphorylation, and secretory pathways, but it can also limit ICD by degrading DAMPs, antigenic cargo, and major histocompatibility complex (MHC) molecules. The net outcome is highly context-dependent and determined by the tumor type, the nature and intensity of the stress, and the level at which autophagy is modulated. Herein, we summarize how autophagy affects the three canonical ICD-associated DAMPs, highlight solid-tumor models in which autophagy supports ICD, and contrast them with systems wherein autophagy inhibition is required for immunogenicity. We then focus on hematological malignancies, especially multiple myeloma, where recent reports implicate the autophagy-related protein GABARAP in bortezomib-induced ICD. Finally, we discuss the translational implications, including rational combinations of autophagy modulators with ICD-inducing chemotherapies, targeted drugs, and cellular immunotherapies, and outline the remaining challenges for safely harnessing the autophagy–ICD axis in the clinical setting.

## Simple Summary

Immunogenic cell death (ICD) is a form of regulated cell death that could change a “cold” tumor into an immune-inflamed “hot” tumor by exposing and releasing damage-associated molecular patterns (DAMPs). Recent works indicate that autophagy can either facilitate or inhibit ICD, depending on the context and which step of the pathway is targeted. In this review, we summarize the current knowledge on the autophagy–ICD axis in various kinds of cancer, and we then focus on hematological malignancies, especially multiple myeloma, in which autophagy and ICD play important roles. We propose how the phase-specific modulation of autophagy could be exploited to design novel immunogenic chemotherapy combinations and improve cellular immunotherapies.

## 1. Introduction

The success of immune checkpoint blockade and cellular immunotherapies has highlighted the importance of pre-existing and treatment-induced antitumor immunity. However, many solid and hematologic malignancies remain immunologically “cold”, with low T-cell infiltration and poor responsiveness to current immunotherapies. One promising strategy to overcome this limitation is to induce immunogenic cell death (ICD), a form of regulated cell death that is sufficient to prime de novo antitumor responses in patients’ bodies after anti-cancer [1,2].

ICD is defined functionally by its ability to vaccinate the host with dying tumor cells in the bodies of patients after anticancer therapies, which suppresses the regrowth of tumor cells, and mechanistically by coordinated DAMP emission. The hallmark DAMPs include the following: (i) the pre-apoptotic translocation of CALR to the cell surface; (ii) active ATP secretion through pannexin-1 channels; (iii) the late release of HMGB1 and other nuclear proteins. These signals promote dendritic-cell (DC) recruitment, antigen uptake, maturation, and cross-presentation, culminating in cytotoxic T-cell priming [1,2,3].

Autophagy is a catabolic process in which cytoplasmic components are sequestered into double-membrane autophagosomes that fuse with lysosomes for degradation [4]. In cancer, autophagy plays a dual role: it can suppress tumor initiation by limiting genomic damage, but it often supports established tumors by buffering metabolic and therapeutic stress [4,5]. Autophagy also affects the tumor microenvironment (TME) by controlling cytokine secretion, antigen presentation, and immune checkpoint expression [6,7].

Recent works suggest that ICD and autophagy are tightly intertwined [8,9,10,11]. The phosphorylation of eIF2α—a central event in the integrated stress response and ICD—is also a hallmark of autophagy induction [8]. Depending on the context, autophagy may promote DAMP exposure and secretion, or it may remove DAMPs and antigenic cargo, rendering cell death non-immunogenic [9,10,11]. This review synthesizes the current knowledge on this autophagy–ICD crosstalk in various types of cancers and explores the translational implications with an emphasis on multiple myeloma.

## 2. Molecular Basis of Immunogenic Cell Death

### 2.1. Canonical DAMPs in ICD

**Figure 1:**
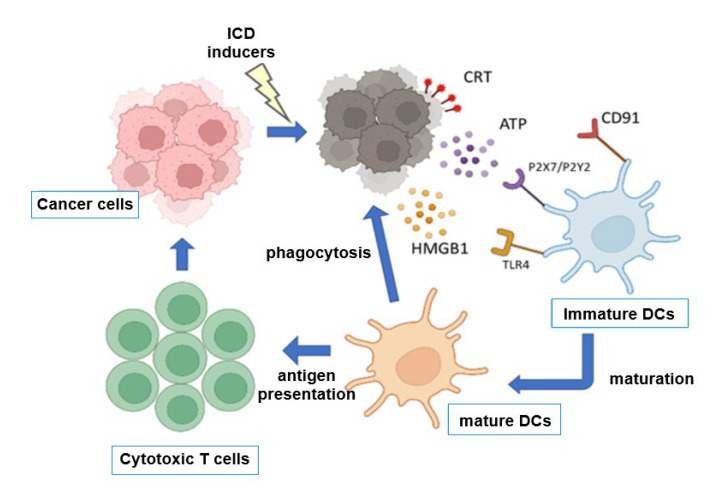
The mechanism of ICD. Treatment of cancer cells by ICD inducers, such as chemotherapeutic drugs or radiotherapy, causes the cell surface expression of calreticulin (CRT) and the secretion of ATP and HMGB1, whose receptors, CD91, P2X7/P2Y2, or TLR4, are expressed on dendritic cells (DCs). Mature DCs can present antigens to cytotoxic T cells and activate these T cells, which can eradicate the remaining cancer cells.

The current consensus definition of ICD centers on the temporally ordered emission of DAMPs, which are mainly CALR, ATP, and HMGB1 [1,2,3]. CALR exposure is enabled by ER stress followed by the unfolded protein response (UPR) in the setting of anthracycline or oxaliplatin treatment [2]. ATP release relies on secretory pathways and serves as a “find-me” signal that recruits and activates purinergic receptor-expressing DCs. HMGB1 release occurs during secondary necrosis and binds TLR4 and other pattern recognition receptors on myeloid cells, licensing efficient antigen presentation and supporting the initiation of adaptive antitumor immunity (Figure 1).

### 2.2. Signaling Pathways Related to ICD Induction

The induction of immunogenic cell death (ICD) is intricately governed by the UPR, especially through the module controlled by eIF2α. This pathway serves as a critical nexus, sensing intracellular damage and translating it into immune-stimulatory signals. The regulators of these pathways include specific eIF2α kinases, such as PERK (EIF2AK3), which acts as an apical sensor of endoplasmic reticulum (ER) stress. Upon activation, the kinase phosphorylates eIF2α, leading to a global attenuation of protein translation [2].

Crucially, this translational arrest is not merely a cytostatic response but a prerequisite for ICD. The cessation of protein synthesis liberates chaperones, such as heat shock proteins 70 and 90 (HSP70/90), from their nascent polypeptide-folding duties. These “freed” chaperones are then recruited to facilitate the translocation of CALR from the ER lumen to the plasma membrane, a hallmark “eat-me” signal of ICD [3]. Concurrently, phosphorylated eIF2α promotes the selective translation of Activating Transcription Factor 4 (ATF4), which transcriptionally upregulates key components of the autophagy machinery (e.g., *ATG5*, *BECN1*) [4]. Therefore, eIF2α phosphorylation integrates stress responses that trigger both autophagy and ICD [12].

## 3. Autophagy Pathways at the Cell Stress–Immunity Interface

### 3.1. Overview of Autophagy in Cancer

**Figure 2:**
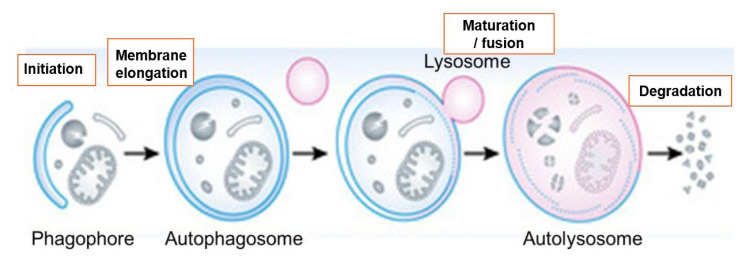
**Schematic overview of autophagic flux.** The process consists of four distinct stages: (1) **Initiation**: Upon cellular stress (e.g., starvation), the phagophore assembly site is nucleated via the ULK1 and Class III PI3K complexes. (2) **Membrane elongation**: The phagophore expands to sequester cytoplasmic cargo, a process driven by the conjugation of LC3-I to phosphatidylethanolamine (PE) to form LC3-II. (3) **Maturation and fusion**: The completed autophagosome fuses with a lysosome to form an autolysosome. (4) **Degradation**: Acidic hydrolases within the autolysosome break down the sequestered cargo, recycling nutrients back into the cytoplasm.

Autophagy proceeds through the initiation, nucleation, elongation, and closure of autophagosomes, followed by fusion with lysosomes (Figure 2).

Core proteins include ULK1, Beclin-1, class III PI3K complexes, ATG conjugation systems, and ATG8/LC3/GABARAP family members. In cancer, autophagy supports survival under nutrient deprivation and hypoxia, maintains mitochondrial quality, and confers resistance to multiple therapies [4].

To examine the role of autophagy in cancer cells, several methods have been used to inhibit each stage of autophagy in experimental models (Table 1), which will be discussed later.

### 3.2. Autophagy in the Tumor Microenvironment

Autophagy modulates antigen presentation by controlling MHC-I and MHC-II trafficking and funneling intracellular antigens into the cross-presentation pathway [13,14]. In DCs, basal autophagy supports antigen processing, whereas excessive autophagy can degrade engulfed antigens before cross-presentation. In tumor cells, autophagy can either display neoantigens via “autophagy-dependent cross-presentation” or sequester them away from MHC loading [14].

Therefore, autophagy could be a central determinant of whether a tumor microenvironment is “cold” or “hot”: secretory autophagy can promote DAMP release and chemokine secretion, while degradative autophagy may remove DAMPs and downregulate MHC-I, thereby limiting T-cell recognition.

## 4. Dual Roles of Autophagy in Immunogenic Cell Death

Autophagy and immunogenic cell death (ICD) are closely intertwined; however, the literature reports both immunosuppressive and immunostimulatory roles for autophagy in the regulation of ICD. Accordingly, we summarize evidence supporting each scenario and discuss the underlying mechanisms that may account for these apparently divergent outcomes in this section.

### 4.1. Autophagy as a Facilitator of ICD

Several studies indicate that intact autophagy is required for ICD induction in cancer cells [8,9,10,11,15,16]. In this setting, the genetic deletion of autophagy genes or pharmacologic inhibition in the early stages of autophagy, such as siRNA against *ATG5*, *ATG7*, and *BECN1*, or 3-Methyladenine, often reduces extracellular ATP levels, dampens CALR exposure, or impairs the cross-presentation of antigens to cytotoxic T cells (Table 2).

### 4.2. Autophagy as an ICD Brake

The enhancement of autophagy has historically been described as permissive or required for specific hallmarks of immunogenic cell death (ICD), most notably ATP secretion (Table 2). However, accumulating evidence indicates that autophagy inhibition—particularly in the late stages—can also facilitate ICD and antitumor immunity by limiting the lysosomal degradation of immunogenic signals. This enhancement is driven by two principal mechanisms: (1) the prevention of MHC-I and tumor antigen degradation, and (2) the amplification of lysosomal stress leading to increased DAMP release.

#### 4.2.1. Restoration of Antigen Presentation and MHC-I Stability

The most critical mechanism identified in recent years is the role of autophagy in degrading MHC class I molecules (MHC-I), thereby contributing to immune evasion. Yamamoto et al. provided landmark evidence in pancreatic cancer, demonstrating that the autophagy cargo receptor NBR1 targets MHC-I for lysosomal degradation [13]. Others have also demonstrated that the genetic or pharmacological inhibition of autophagy restores surface MHC-I levels and facilitates CD8+ T-cell recognition [22,23,24,25].

#### 4.2.2. Amplification of ATP Secretion and DAMP Release

Late-stage autophagy inhibition—typically via lysosome-targeting agents that block autophagosome clearance and impair lysosomal function—can amplify lysosomal stress by driving lysosome swelling, defective degradation, and membrane permeabilization. Chloroquine accumulates in lysosomes, increases the lysosomal volume, and destabilizes lysosomal and plasma membranes, promoting necrosis-like death; this membrane integrity loss facilitates the passive release of DAMPs, such as HMGB1 [26]. In contrast, ATP release during “classical” ICD is a regulated process that requires pre-mortem autophagy to maintain lysosomal ATP stores and execute LAMP1/PANX1-dependent lysosomal exocytosis; therefore, late-stage blockade may increase lytic DAMP emission yet blunt regulated ATP export, depending on the context and death modality [27].

Taken together, the inhibition of autophagy (mainly end-stage) serves as a powerful strategy to enhance antitumor immune responses (Table 3).

### 4.3. Determinants of the Autophagy–ICD Outcome

**Figure 3:**
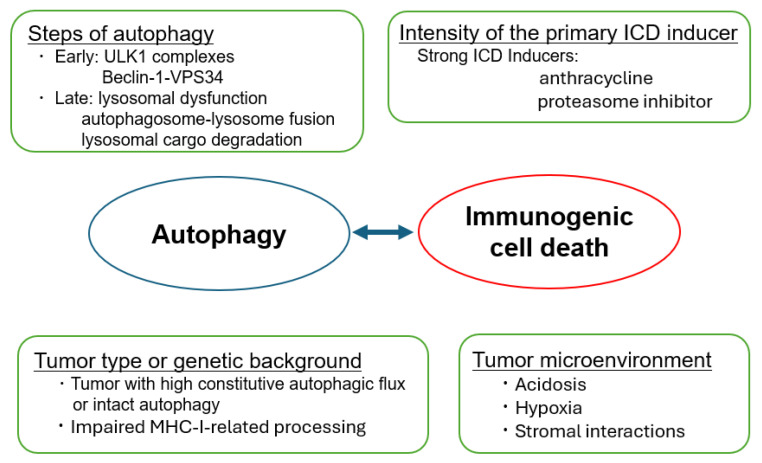
Determinants of the autophagy–ICD outcome. The autophagy–ICD axis can be summarized via the following conceptual frameworks, including the stages of autophagy, intensity of the primary ICD inducer, tumor type or genetic background, and tumor microenvironment.

As outlined above, autophagy can either promote or suppress ICD. The autophagy–ICD axis can be largely distilled into the following conceptual frameworks (Figure 3).

#### 4.3.1. Steps of Autophagy

Early autophagy inhibition at the initiation/nucleation stage—targeting, for example, ULK1 complexes or the Beclin-1–VPS34 axis—frequently attenuates key ICD outputs, notably extracellular ATP release and, in some settings, CALR exposure on the tumor cell surface, thereby dampening dendritic-cell recruitment and maturation as well as the subsequent T-cell priming. Mechanistically, this is consistent with the close coupling between ER stress signaling (including eIF2α phosphorylation) and the coordinated execution of both autophagic programs and ICD hallmarks, such that interrupting early autophagy can disrupt the vesicular trafficking and regulated secretory routes required for optimal “adjuvanticity”. In contrast, late-stage autophagy blockade—through lysosomal dysfunction, impaired autophagosome–lysosome fusion, or defective lysosomal cargo degradation—tends to promote the intracellular accumulation of damaged organelles and undegraded cargo, thereby increasing the availability of DAMP reservoirs and favoring their leakage or release under stress conditions.

#### 4.3.2. Intensity of the Primary ICD Inducer

The qualitative nature and quantitative intensity of the initiating drugs are major determinants of whether autophagy is dispensable, supportive, or even antagonistic to ICD. Potent ICD inducers that elicit robust ER stress and oxidative damage—such as anthracyclines or proteasome inhibitors—can drive a sufficiently strong danger-signaling program (e.g., integrated stress responses and inflammatory transcriptional outputs) to preserve core ICD features even when the autophagic capacity is partially compromised. By contrast, when the initiating stress is comparatively mild or chronic, intact autophagic machinery may become rate-limiting for effective DAMP emission, including regulated ATP release and the coordinated maturation/trafficking of immunostimulatory signals that condition dendritic-cell uptake and priming. Therefore, understanding the strength and point in the pathway of the ICD inducer is important to determine immunogenicity when combining ICD inducers and autophagy modulators.

#### 4.3.3. Tumor Type and Genetic Background

Tumor-intrinsic baseline autophagy and the integrity of antigen presentation machinery are key variables that affect how cancer cells execute ICD and how the immune system perceives them. Tumors with high constitutive autophagic flux (often reflecting chronic proteotoxic, metabolic, or hypoxic stress) may buffer therapy-induced danger signaling by rapidly clearing damaged organelles and stress-associated macromolecules, thereby limiting the magnitude or duration of DAMP emission and inflammatory cues. Conversely, in settings where ICD relies on the regulated secretion and vesicular trafficking of immunostimulatory signals, an intact autophagy program may be required to sustain ATP release and other adjuvanticity features, particularly in “cold” microenvironments in which the immune priming is already suboptimal [17,18]. Moreover, genetic or functional defects in antigen presentation pathways (e.g., impaired MHC-I–related processing/loading) can decouple DAMP emission from effective immune recognition, even when autophagy-dependent stress responses and ICD hallmarks are triggered. Reduced MHC expression can blunt dendritic-cell cross-priming and T-cell activation [38,39]. Thus, the baseline autophagy state and antigen presentation competence should be considered jointly when interpreting ICD phenotypes and designing rational combinations that convert immunologically silent death into productive antitumor immunity.

#### 4.3.4. Microenvironmental Factors

Acidosis, hypoxia, and stromal interactions can substantially rewire the autophagy dependence of ICD, thereby shifting whether autophagy functions as a facilitator of DAMP emission or as an adaptive program that attenuates immune recognition instead.

Norcantharidin (NCTD) induces ICD in bladder cancer cells in acidic conditions, increasing the cell surface expression of CALR and promoting DC maturation. In one study, these immunogenic outputs were autophagy-dependent, as the pharmacologic autophagy inhibition abrogated ecto-CALR exposure and DC maturation. Notably, the DC maturation phenotype differed between acidic and physiological pH conditions [40].

Hypoxia provides a complementary axis through which the microenvironment can bias the outcome: in hypoxic tumors, the activation of the PERK arm of the UPR upregulates autophagy, which subsequently downregulates the surface MHC class I expression and thereby suppresses antigen presentation [41]. Finally, stromal crosstalk can determine whether targeting autophagy enhances or undermines immunity because autophagy in non-malignant compartments actively shapes the inflammatory tone. In pancreatic cancer models, the autophagy of cancer-associated fibroblasts (CAFs) affects the immunochemotherapy responsiveness, suggesting that microenvironmental autophagy (beyond tumor cells) is also a key determinant of immune outcomes [42].

These findings support a model in which the immunological consequences of autophagy are dictated by its timing, magnitude, and surrounding environment, and this context dependence has direct therapeutic implications. For example, inhibiting late-stage autophagy with strong ICD inducers is one suitable treatment option for obtaining durable antitumor immunogenicity. On the other hand, autophagy may support tumor survival under hypoxia/therapy stress and can reduce antigen presentation via MHC-I degradation. Thus, autophagy modulation may inadvertently foster immune escape or tumorigenesis; biomarker-guided selection and rational combinations are essential.

## 5. Lessons from Hematologic Malignancies, Including Multiple Myeloma

### 5.1. ICD in Hematologic Cancers

Although much of the ICD framework was initially established in solid tumors, such as colon, breast, and lung cancers, the underlying principles are not tissue-restricted: dying malignant cells can emit DAMPs and tumor antigens that shape antigen presentation and downstream T-cell responses. Preclinical and early clinical evidence have shown that hematologic malignancies, including leukemia, lymphoma, and myeloma cells, can also undergo ICD upon exposure to chemotherapies and selected targeted agents [43,44,45]. In fact, ICD-related gene signatures correlate with prognoses and immune infiltration in several hematologic malignancies [46,47].

Multiple myeloma especially offers a complementary and clinically relevant setting to interrogate this biology because the bone marrow niche is strongly immunomodulatory and standard-of-care therapies and immunotherapies (e.g., proteasome inhibitors, monoclonal antibodies, cellular therapies) provide tractable contexts in which ICD-associated signals and autophagy-dependent pathways can be linked to immune activation or escape [48,49,50]. We have also reported that bortezomib and carfilzomib could induce ICD via ER stress induction and the subsequent UPRs [51]. However, the comprehensive mapping of the autophagy–ICD interplay is still limited compared with that of solid tumors.

### 5.2. GABARAP-Dependent Autophagy and Bortezomib-Induced ICD in Multiple Myeloma

Multiple myeloma (MM) is highly dependent on proteostasis because of its intense immunoglobulin synthesis. Proteasome inhibitors (PIs) such as bortezomib induce ER stress and can elicit ICD in MM cells, contributing to their clinical activities [52,53].

Recently, Gulla et al. provided mechanistic insight into how autophagy shapes PI-induced ICD in MM. Gamma-aminobutyric acid receptor-associated protein (GABARAP)-deficient myeloma cells showed reduced CALR exposure and ATP release despite undergoing cell death [54]. The involvement of autophagy-mediated Golgi restoration in this attenuation of ICD has been suggested; however, the detailed mechanism remains unclear. Treatment with rapamycin, the autophagy inducer, restored CALR exposure, suggesting that the early stage of autophagy plays some role in ICD in the myeloma setting. The authors also proposed that GABARAP could serve as a biomarker for selecting patients who are likely to benefit from ICD-inducing chemotherapies.

### 5.3. Late-Stage Autophagy Inhibition in Multiple Myeloma

While early-stage autophagy deficiency can impair ICD in MM, late-stage pharmacologic inhibition may have a different effect. Ambroxol, a mucolytic agent widely used in respiratory medicine, was recently identified as a late-stage autophagy inhibitor with anti-myeloma activity [52]. In myeloma cell lines, ambroxol treatment led to autophagosome and LC3-II accumulation, with impaired autophagic degradation, consistent with blockade at the autophagosome fusion or lysosomal function step. Moreover, synergistic anti-myeloma effects were observed when combined with histone deacetylase (HDAC) inhibitors, with a favorable toxicity profile in preclinical models [55]. Although further evaluation should be conducted, this late-phase autophagy inhibition might be exploited to amplify ICD in MM.

### 5.4. Future Strategy to Utilize Autophagy-Modulating Drugs to Strengthen ICD in Multiple Myeloma

**Figure 4:**
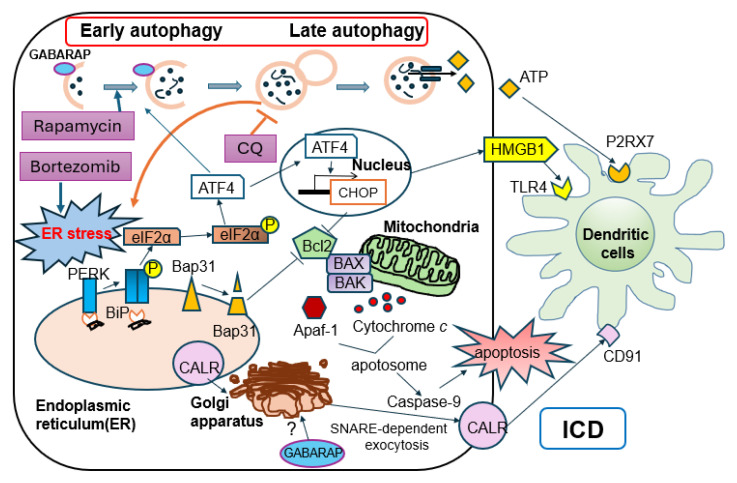
Effects of bortezomib-induced ICD and late-stage autophagy inhibition by chloroquine (CQ), or early-stage autophagy activation by Rapamycin in myeloma cells. Bortezomib causes ER stress, which triggers ICD signals, such as PERK-eIF2α axis, facilitating CALR exposure on the cell surface and HMGB1 release, leading to dendritic-cell maturation. Although chloroquine blocks late-stage autophagy and can reduce autophagy-dependent ATP release, it also prevents immunoglobulin degradation, increasing proteotoxic stress and further enhancing ER stress, thereby strengthening ICD overall. GABARAP may normally act at the Golgi apparatus to facilitate SNARE-dependent exocytosis and promote the translocation of CALR to the cell surface. In GABARAP-deficient cells, Rapamycin may restore ATP secretion by stimulating early-stage autophagy or facilitate CALR exposure by acting on the Golgi apparatus.

As mentioned above, compared with solid tumors, multiple myeloma is exposed to excessive ER stress driven by malignant immunoglobulin production, which may render it more prone to ICD. The key treatment drugs include proteasome inhibitors, which facilitate ER stress and eIF2α activation within the PERK arm, which could also promote autophagy via the downstream transcription factor ATF4 [56]. Integrating prior reports and observations from solid tumors, a potential therapeutic strategy in myeloma would be to enhance ICD through the context-dependent modulation of autophagy in addition to the ICD inducer bortezomib: in patients with low GABARAP expression, combining an early-stage autophagy activator such as rapamycin may restore autophagic competence and thereby augment ICD, whereas in patients with preserved GABARAP expression, autophagy inhibitors such as chloroquine or ambroxol could be employed to potentiate ICD (Figure 4). Moreover, beyond GABARAP, it will be important to identify additional determinants that modulate autophagy, and to carefully evaluate whether an autophagy activator or a late-stage autophagy inhibitor should be combined. Collectively, these considerations raise the possibility of a new, personalized treatment approach that leverages autophagy modulation to maximize ICD efficacy in multiple myeloma.

## 6. Translational Implications

### 6.1. Rational Combinations with Anticancer Agents

Accordingly, combining ICD-inducing anticancer agents with autophagy modulators is expected to amplify antitumor immunity by enhancing antigen presentation and effector immune-cell recruitment, thereby improving therapeutic efficacy for cancers.

However, rational designs should prioritize mechanistically defined pairings (e.g., selective late-stage/lysosomal blockade only when it increases DAMP/antigen presentation without boosting stress tolerance) and monitoring (tumor growth kinetics, MHC-I expression, and immune infiltration) and be tested in in vivo immunocompetent models to exclude pro-tumorigenic effects before clinical translation.

Based on these considerations, we propose several therapeutic strategies.

#### 6.1.1. Autophagy Modulation to Rescue “Weak ICD”

For drugs that induce subthreshold ER stress or DAMP emission, autophagy induction (e.g., by autophagy-amplifying nanoparticles) might enhance immunogenicity, particularly in solid tumors where degradative autophagy is not overly dominant [53]. However, it should be noted that heightened autophagy may also facilitate cancer cell proliferation, and this possibility warrants careful consideration.

#### 6.1.2. Late-Stage Autophagy Inhibition to Prevent DAMP Degradation

In settings with strong ER stress and high basal autophagy—such as MM treated with PIs—late-stage inhibitors, such as chloroquine derivatives or more selective lysosomal inhibitors, may expand the DAMP-emitting compartments and prolong the antigen availability for DCs. Clinical translation will require careful dosing to avoid systemic toxicity and unwanted immunosuppression.

### 6.2. Synergy with Checkpoint Blockade and CAR-T/TCR-T

ICD inducers can increase antigen presentation and T-cell infiltration, thereby sensitizing tumors to checkpoint blockade [57,58]. Enhancing ICD along with autophagy modification could also improve the depth and durability of responses to PD-1/PD-L1 blockade by increasing antigen loads and inflamed TME signatures [59,60]. In addition, the effects of CAR-T therapies can be increased by targeting autophagy and potentially modulating the TME to favor CAR-T expansion and persistence [61,62].

However, autophagy also supports T-cell metabolism and memory; global inhibition may impair effector and memory T-cell function [63]. Therefore, tumor-selective or transient autophagy modulation—using drug scheduling, antibody–drug conjugates, or nanoparticle delivery—will be essential.

## 7. Conclusions

Autophagy and ICD are closely related processes that collectively determine whether dying tumor cells act as an immunological vaccine. Experimental evidence from solid tumors and hematologic malignancies, including multiple myeloma, reveals that autophagy can both promote and restrain ICD in a phase- and context-dependent manner. Early-stage autophagy competence, exemplified by GABARAP, appears necessary for bortezomib-induced ICD in myeloma, whereas late-stage autophagy inhibition by agents such as chloroquine amplifies ICD-like death by preventing MHC or DAMP degradation.

These insights suggest that precision targeting of the autophagy–ICD axis could transform currently “cold” tumors into “hot” ones and improve the efficacy of chemotherapy, targeted therapies, checkpoint blockade, and cellular immunotherapies. The mechanistic dissection of autophagy–ICD crosstalk and biomarker-guided clinical trials should be combined in future translational studies.

## Figures and Tables

**Table 1:** Inhibitors of autophagy stages.

Steps	Related Molecules	Inhibitors
Initiation	ULK1, Beclin-1	3-MA, Wortmannin
Membrane elongation	ATGs, LC3/GABARAP family members	Genetic modulation (siRNA)
Maturation/fusion	Rab7, SNARE complex, LAMP1/2	Bafilomycin A1, CQ, HCQ
Degradation	SQSTM1	E64d, Pepstatin A

**Abbreviations:** ULK1: Unc-51 like autophagy activating kinase 1; Beclin-1: Beclin 1 (autophagy-related 6); ATG: autophagy-related; LC3: microtubule-associated protein 1 light chain 3; GABARAP: GABA type A receptor-associated protein; Rab7: Ras-related protein Rab-7; SNARE: soluble N-ethylmaleimide-sensitive factor attachment protein receptor; LAMP1/2: lysosomal-associated membrane protein 1/2; SQSTM1: Sequestosome 1; 3-MA: 3-Methyladenine; CQ: chloroquine; HCQ: hydroxychloroquine; E64d: E-64-derived compound.

**Table 2:** Representative studies linking **autophagy pathway inhibition (early stage)** to **immunogenic cell death (ICD) hallmarks** and/or **tumor immunogenicity** (immune inactivation readouts) in defined cancer types.

Cancer Type	Autophagy Inhibition Method	ICD Markers	Key Mechanism	Reference
Colon	siRNA (*ATG5*, *ATG7*), Baf, HCQ,	ATP Secretion	Autophagy inhibition reduced mitoxantrone- or oxaliplatin-induced ATP secretion.	[17]
Osteosarcoma	siRNA (*ATG5*)	ATP Secretion	siRNA-mediated depletion of *ATG5* blocked ATP secretion induced by oxaliplatin.	[18]
Lung	siRNA *(BECN1*, *ATG5*)	ATP Secretion	Genetic depletion of *ATG5* and *Beclin 1* reduced ATP secretion and suppressed radiotherapy efficacy.	[19]
Pancreatic	siRNA (*ATG5*, *ATG7*), Baf	HMGB1 Release	Inhibition of autophagy blocked ferroptosis activator-induced HMGB1 release.	[20]
Colorectal	-	HMGB1 Release, ATP Secretion	PDT amplified oxaliplatin-induced ICD along with autophagy.	[21]

**Abbreviations:** Baf: Bafilomycin A1; ATG: autophagy-related; ATP: adenosine triphosphate; HMGB1: high-mobility group box-1; Beclin-1: Beclin 1 (autophagy-related 6); ICD: immunogenic cell death.

**Table 3:** Representative studies linking **autophagy pathway inhibition** to **immunogenic cell death (ICD) hallmarks** and/or **tumor immunogenicity** (immune activation readouts) in defined cancer types.

Cancer Type	Inhibition Strategy	ICD Markers	Key Mechanisms	Reference
Colon	CQ, siRNA (Atg5)	CALR exposure, ATP release	Co-delivery of oxaliplatin; CQ prevents late autophagy, amplifying CALR exposure.	[28]
Pancreatic	CQ, genetic (Nbr1 KO)	MHC-I surface levels, CD8+ T-cell accumulation	NBR1-mediated selective autophagy degrades MHC-I; inhibition restores antigen presentation and cytotoxicity.	[13]
Neuroblastoma	CQ	MHC-I expression, tumor infiltration	Autophagy inhibition with CQ boosts antitumor immune responses of anti-GD2 antibody.	[29]
Melanoma	CQ	CALR exposure, HMGB1 release	Combination with checkpoint inhibitors; autophagy inhibition converts “cold” tumors to “hot” via increased immunogenicity.	[30]
Colon	CQ	CALR exposure, HMGB1 release CD8+ T-cell accumulation	Liposomes loaded with copper peroxide and CQ enhance the immunogenicity of cell death.	[31]
Lung	CQ	HMGB1 release CD8+ T-cell accumulation	Mild photothermal therapy with CQ increased DAMP release and CD8+ T-cell accumulation.	[32]
Colon	CQ	DC maturation T-cell response	Combination of 5-FU and CQ increased DC maturation and stimulated T-cell responses.	[33]
Breast	CQ	CALR exposure, HMGB1 release	Photodynamic therapy with fucoidan (Fuc)-based chlorin e6 (Ce6)–CQ hydrogels enhanced ICD.	[34]
Colon	Cepharanthine	CALR exposure, DC activation	Autophagy inhibition by cepharanthine induced immunogenic cell death markers.	[35]
Colon	HCQ	CALR exposure, ATP release	Shikonin with encapsulate HCQ promotes ICD.	[36]
Breast	CQ	CALR exposure, ATP release	ER-targeted PDT combined with CQ synergistically induces ER stress and drives robust ICD via autophagy inhibition.	[37]

**Abbreviations:** ICD: immunogenic cell death; CALR: calreticulin; DC: dendritic cell; CQ: chloroquine; HCQ: hydroxychloroquine.

## Data Availability

No new data were created or analyzed in this study. Data sharing is not applicable.

## Abbreviations

The following abbreviations are used in this manuscript:

ATF4	Activating transcription factor 4
ATG	Autophagy-related
ATP	Adenosine triphosphate
CALR	Calreticulin
DAMPs	Damage-associated molecular patterns
DC	Dendritic cell
eIF2α	Eukaryotic translation initiation factor-2α
ER	Endoplasmic reticulum
GABARAP	GABA type A receptor-associated protein
HDAC	Histone deacetylase
HMGB1	High-mobility group box-1
HSP	Heat shock protein
ICD	Immunogenic cell death
IFN	Interferon
LC3	Microtubule-associated protein 1 light chain 3
MHC	Major histocompatibility complex
MM	Multiple myeloma
PDT	Photodynamic therapy
PERK	Protein kinase R-like endoplasmic reticulum kinase
PI3K	Phosphoinositide 3-kinase
PIs	Proteasome inhibitors
ROS	Reactive oxygen species
STING	Stimulator of interferon genes
TME	Tumor microenvironment
UPR	Unfolded protein response
